# Genito-Urinary and Syphilitic Diseases

**Published:** 1902-09

**Authors:** Byron H. Daggett

**Affiliations:** Buffalo, N. Y.


### Genito-Urinary and Syphilitic Diseases.

CONDUCTED BY BYRON H. DAGGETT, M. D., Buffalo, N. Y.

URIC ACID: ITS SOURCES AND EFFECTS.

JAMES TYSON (*New York Med. Jour.*, March 15, 1902.) The only form in which uric acid can exist in the blood is in the shape of a quadriurate. (Roberts Croonian lectures.)

The quadriurate is a very soluble, unstable combination with sodium. This compound holds the uric acid in the blood fluids of the body. It reacts readily with the sodium carbonates of the blood, forming a biuret, which is not easily soluble and which sometimes deposits in needlelike crystals in the cartilages, ligaments and fibrous tissues of the body. This compound has been found in abundance in the blood during gouty seizures, in chronic nephritis, anemias and leucocytosis.

Urea is said to be the ultimate product of nitrogenous metabolism and its remote source is the food albumins and to a less extent the fixed albumin of the tissues. Some observers consider that uric acid is formed out of urea in the kidneys.

Zalesky, Levison and Kolisch regard the kidneys as important uric acid forming organs. Urea is formed by the amide-acids into which albumins are converted during tryptic digestion and putrefaction and by the action of acids and alkalies. The larger part is formed from the ammonium salt of paralactic acid in which albumin nitrogen is set free. A fermentative, synthetical process forms urea from the paralactate and carbonate of ammonium. Urea also is formed from the albumin by hydrolysis. Drechel says that 10 per cent. of the urea is thus formed. The principal source of urea is paralactic acid, and the liver is the chief seat of its formation, although it is formed synthetically in other organs.

Whence comes uric acid?

Uric acid is one of the end products of animal metabolism and is remotely derived from ingested food. Modern study indicates that uric acid in man is derived from the nucleins of ingested food, from the body cells, especially leucocytes, the change taking place in all the organs of the body, particularly in the spleen and lymphatic glands. It is likely that uric acid is formed from disintegrating cells in every part of the body, most abundantly in the glands and lymphatics.

*Its Clinical and Pathological Significance.*—Uric acid is not poisonous, the harm it does is local: (1) it is deposited in the kidney and urinary passages, forming calculus and gravel; (2) it is deposited in the joints as a biurate; (3) uric acid accumulates in the blood (as a biurate) in gout, chronic lead poisoning, chronic nephritis, leucemia and pernicious anemia.

How does uric acid get into the situations named? Is it harmless? If so, what agent produces the symptoms associated with its presence?

The formation of calculus and gravel is the easiest explained of all the clinical facts. It comes from the kidney and is precipitated from the urine by an over saturation of the urine with the uric acid and its salts, especially the biurate. Scanty and high specific gravity of the urine are found where such deposits occur. The production of uric acid is not necessarily increased, the quantity of water is greatly reduced, so that there is not enough to hold it in solution.

How is it deposited in the fibrous tissues?

There may be an abnormal quantity of the biurate in the blood. A stasis of the lymph current favoring precipitation.

The recent views held as to the rôle played by uric acid limit its harmfulness to the formation of concretion and credit the alloxuric basis with the harmful phenomena of the so-called uric acid lesions; that the formation of uric acid is beneficial and not baneful. It deposits with insufficient solvent.

*Treatment.*—Abundance of alkaline or plain water, especially between meals, and the exclusion of proteid foods to a degree sufficient to eliminate uric acid from the urine, together with a liberal amount of out-door exercise, is the treatment for all uric acid manifestations. The best treatment for articular deposits and swellings is massage and hot baths.

Treatment recommended by Crofton: there are two indications (1) a reduction in the nuclein catabolism; (2) a raising of the processes of oxygenation. To attain the first object avoid everything that produces leucocytosis, such as quinine, pilocarpine, atropine and proteid foods. Overeating should be forbidden.

In a true uric acid case there will be excessive nuclein-catabolism despite all we may be able to do, in the very nature of the taint, and restrictions in diet will not be of permanent benefit; the chief point of attack will be in the direction of raising oxidation. A uric acid case should be treated as an anemic case in all measures employed to promote the oxygenation powers of the blood, *i. e.*, the production of an increase in the red blood corpuscles and of the hemoglobin and its chief oxygen carrier—iron.

Give iron, arsenic and oxygen.

---

### HEMATURIA.

WILLIAM L. OTIS (*New York Med. Jour.*) states that blood in the urine indicates a lesion in the urinary tract—its significance may be trifling or serious. Usually its presence may be determined by the "naked eye" or so scanty as to require other means for identification; one part of blood to 1500 of urine will produce a smoky tint; 1 to 500, a bright cherry red. If a small quantity of liquor potassium be added to the urine and then heated to the boiling point, a bright red or brownish green sediment is formed.

A simple test, not strictly reliable, because other substances besides blood may give reaction, consists in making an emulsion of equal quantities of guaiac and oil of turpentine, the urine carefully added; if blood is present a bright blue precipitate will be formed.

The best and most positive test for blood is the microscope. If the corpuscles have been in the urine but a short time, they will have the appearance of those from the veins. If they have been exposed for some time, they become considerably altered.

In dilute, alkaline urine the corpuscles are soon destroyed. Add a concentrated solution of sulphate of sodium and they become visible again, appearing angular and distorted.

Blood in the urine may be definitely determined by the spectroscope. If blood be found in the urine, the next step is to locate its source. To do this divide the urinary tract into four portions: (1) Meatus to compressor muscle: If the bleeding point is in this division the flow will be continuous, or it will exude from the meatus as there is no obstacle to its exit. Pass the urine in two portions and the first will be stained and the second clear. Bleeding may be located by sectional pressure along the urethra, or its source may be found by the urethrascope; (2) the prostatic or posterior urethra: if the point of hemorrhage is in the prostatic urethra the outflow is checked by the external sphincter and it is passed in urination, mixed urine and blood; if the amount be small it appears at the beginning and the end of urination. If the hemorrhage is profuse it flows backward into the bladder and colors the urine uniformly. The chemical reaction of the urine is not a reliable guide for if the hemorrhage be profuse the blood serum tends to neutralise the acid reaction of normal urine. If the alkalinity be due to ammoniacal fermentation, it has taken place in the bladder, for this condition does not occur in the kidney. If there are no clots in bladder, it is readily freed from renal blood. Fresh bladder hemorrhage is bright red in color; if the stain is due to clots or kidney hemorrhage, it is dark red or brown.

The normal bladder absorbs very slowly, but if there be a lesion of the mucous surface absorption is rapid, so that if a solution of potassium iodide be introduced into the bladder the characteristic reaction of iodine is shown in the saliva. The lesion may be located by the use of the cystoscope, or it may be determined whether the hemorrhage is from the bladder, or either ureter. The trigonum and mouths of the ureters should be carefully examined.

If the bladder and urethra be excluded as the source of the bleeding, then it must be from the superior portion of the urinary tract. Kidney hemorrhage is indicated by the uniformity and darker color of the urine. Kidney hematuria may be intermittent, due to blocking of the ureter by a clot, the other kidney secreting normal urine. This is almost pathognomonic of renal hemorrhage. Increased amount of blood in the morning indicates renal hemorrhage. Blood clots are pathognomonic of renal blood. Long, round clots and casts of the pelvis and calites render the diagnosis absolute. A large kidney or a tumor indicates kidney hemorrhage.

---

HORWITZ (*Jour. Amer. Med. Assn.*, June 21, 1902.) (1) Primary tuberculous infection of epididymis and testicle may and often does occur; (2) infection of the testicle usually secondary to that of the epididymis; (3) these infections take place through the circulation; (4) secondary involvement of the organs may follow from tuberculosis of other organs; (5) invasion of testicle may be acute or chronic, simulating syphilitic or malignant disease; (6) in doubtful cases the tuberculin test should be applied; (7) radical surgery should be applied in the primary form; (8) in doubtful cases with hydrocele the fluid should be examined for tubercle bacilli; (9) in extensive co-existing tuberculous lesion of other organs, radical operations are contra-indicated.

